# Investigating the Link Between Glucagon-Like Peptide-1 (GLP-1) Receptor Agonists and Non-arteritic Ischemic Optic Neuropathy: A Case Report of Semaglutide-Induced Optic Neuropathy

**DOI:** 10.7759/cureus.102472

**Published:** 2026-01-28

**Authors:** Mashair Bakheet, Attiqa Chaudhary

**Affiliations:** 1 Ophthalmology Department, Magrabi Health, Riyadh Hospital, Riyadh, SAU; 2 Medicine Department, Austin Health, Melbourne, AUS

**Keywords:** diabetes mellitus, glp-1 ra, glp-1 receptor agonist, naion, non-arteritic anterior ischemic optic neuropathy, optic neuropathy, semaglutide

## Abstract

Glucagon-like peptide-1 receptor agonists (GLP-1 RAs) are newer medications used to treat type 2 diabetes mellitus (T2DM) and obesity. There is growing evidence of a potential association between the use of GLP-1 RAs and non-arteritic anterior ischemic optic neuropathy (NAION). Here, we report a case of NAION in a young patient with diabetes receiving semaglutide (GLP-1 RA), which showed a complete resolution of symptoms after discontinuing the GLP-1 RA. This case adds to growing concerns, given emerging evidence suggesting an increased incidence of NAION among users of GLP-1 RAs. Further studies are required to clarify causality, identify risk factors, and develop monitoring strategies for patients receiving GLP-1 RAs.

## Introduction

Non-arteritic anterior ischemic optic neuropathy (NAION) is a significant cause of vision loss in adults. In people over 50, it is the most common cause of sudden optic nerve damage after glaucoma. NAION usually leads to sudden, painless vision loss in one eye [1]. It occurs when a brief drop in blood flow, often due to low blood pressure at night or small-vessel disease, damages the front part of the optic nerve. This damage causes swelling and increased pressure, which can worsen the injury. People with a crowded optic disc or a small cup-to-disc ratio are at higher risk for NAION. Other risk factors include high blood pressure, diabetes, high cholesterol, sleep apnea, low nighttime blood pressure, atherosclerosis, and some medications. For example, phosphodiesterase inhibitors may trigger NAION [2]. Glucagon-like peptide-1 receptor agonists (GLP-1 RAs) are newer drugs that lower blood sugar. They help the pancreas release more insulin, reduce glucagon secretion, lower liver glucose production, and slow gastric emptying. GLP-1 RAs also help people feel full and improve how the body uses insulin. They are used to treat type 2 diabetes mellitus (T2DM) and support weight loss. Many studies show that they are safe for the heart and kidneys. Recently, higher doses of semaglutide have been shown to help with weight loss and are now approved for the treatment of obesity [3]. Semaglutide, a potent GLP-1 RA taken once weekly, lowers glycated hemoglobin (HbA1c), body weight, and blood pressure [4]. However, new research has raised concerns about possible eye problems with semaglutide. Some studies have found more cases of diabetic retinopathy in patients using GLP-1 RAs, especially semaglutide [5]. Other studies have also found a higher risk of NAION in patients taking semaglutide compared to those on other diabetes or weight loss treatments [6,7]. This report describes a case of NAION in a patient with diabetes who was using semaglutide injections to manage blood sugar and weight.

## Case presentation

**Figure 1:**
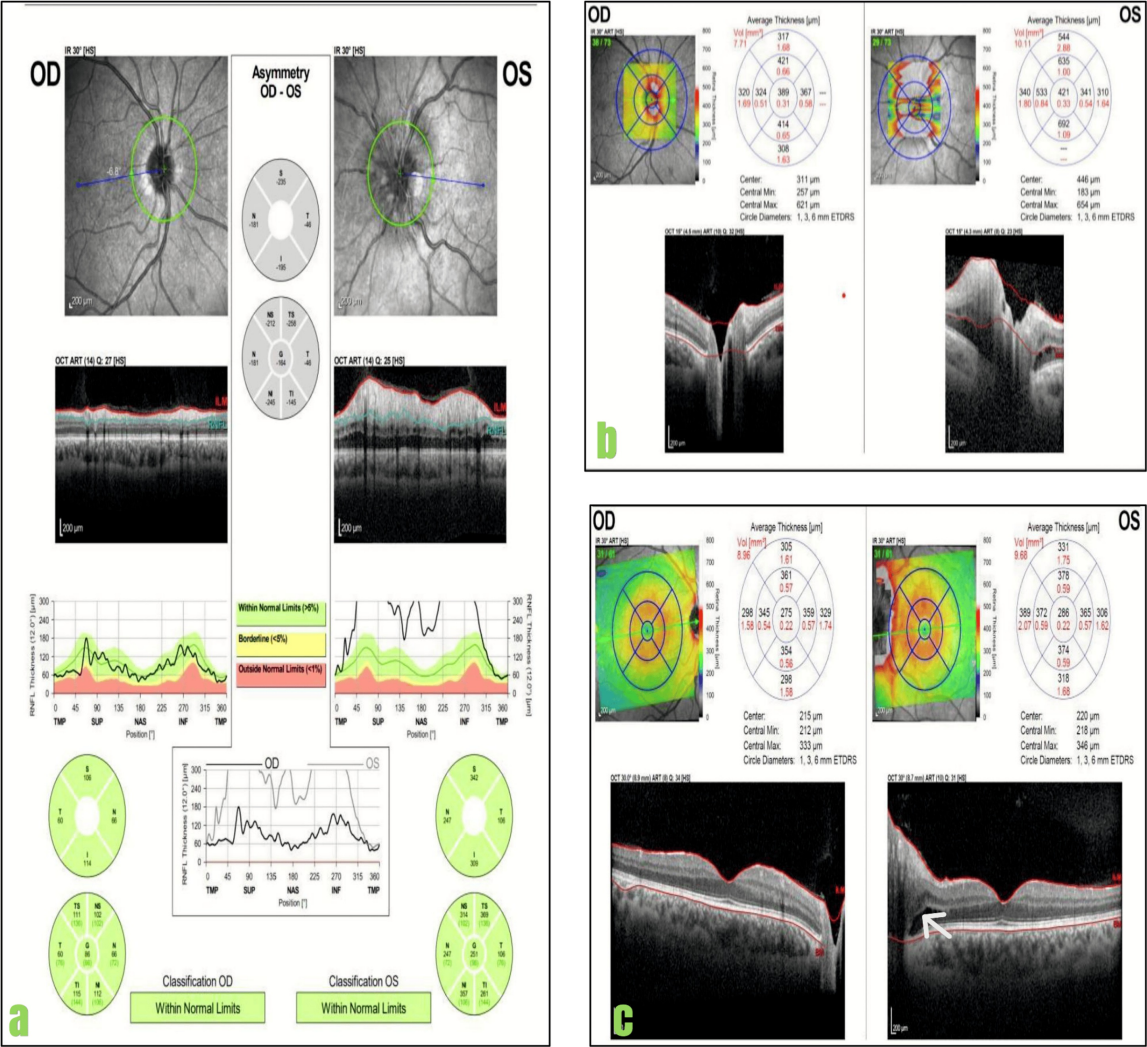
Optical coherence tomography (OCT) of both eyes. Heidelberg OCT images of both eyes show the left eye (OS) with increased retinal nerve fiber layer (RNFL) thickness (a) and increased optic nerve head volume (b) with intraretinal fluid (arrow) in the macular OCT thickness map (c). OD: right eye

**Figure 2:**
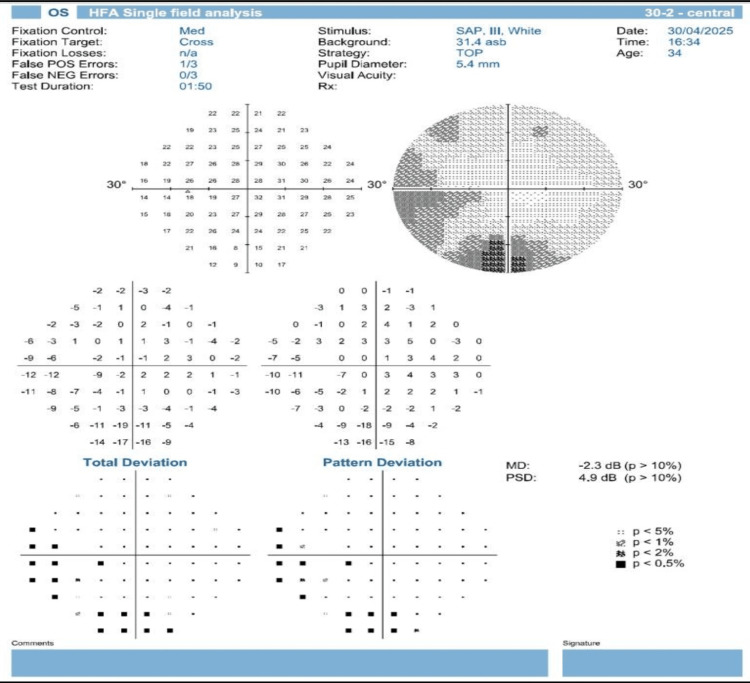
Humphrey’s 30-2 visual fields of the left eye (OS). The left eye demonstrates an inferior partial altitudinal visual field defect with high PSD and mild negative MD. PSD, pattern standard deviation; MD, mean deviation; HFA, Humphrey field analyzer

A 34-year-old gentleman presented to the neuro-ophthalmology clinic with a one-week history of blurred vision in the left eye. He denied any trauma and any previous history of similar complaints. Systemic history revealed that he had been diabetic for 15 years, had a recent change in his medication, and had been using semaglutide injections for the last six months. He was a nonsmoker and denied the use of any recreational drugs. On examination, his blood pressure was 120/75 mmHg. His body mass index (BMI) at presentation was 30.4 kg/m², which had been reduced from 34.5 kg/m² six months after semaglutide initiation. On ocular examination, visual acuity was 20/20 in both eyes with full color vision, but a left relative afferent pupillary defect was observed. Fundus examination revealed bilateral crowded optic discs, with discs’ diameter around 1.4-1.5 mm, and a cup disc ratio of <0.2 in both eyes, and there was left optic disc edema. The patient was emmetropic, with axial lengths of 23 mm in the right eye and 22.8 mm in the left eye. Optical coherence tomography (OCT) was performed, which showed increased retinal nerve fiber layer (RNFL) thickness with intraretinal fluid in the left eye, as shown in Figure 1. Humphrey visual field (HVF) testing was also performed, revealing a left inferior altitudinal defect, as shown in Figure 2. A diagnosis of left‑eye NAION was made based on the findings.

The patient was referred to a neurologist for a workup of optic neuropathy. Complete blood count (CBC), erythrocyte sedimentation rate (ESR), C-reactive protein (CRP), HbA1c, fasting blood glucose, and lipid profile were performed. Infectious, vasculitis, and autoimmune panels were all unremarkable. A magnetic resonance imaging (MRI) of the brain and orbits with contrast was performed, and all were normal. The patient was referred to his endocrinologist. Semaglutide was discontinued, and diabetes and weight management were optimized. At the two-week follow-up, the patient’s symptoms remained unchanged; however, his examination was stable. At the two‑month follow‑up, the patient showed complete recovery of visual function, with the regression of optic disc edema and intraretinal fluid as confirmed by OCT.

## Discussion

Semaglutide, a GLP-1 RA, is a leading treatment for T2DM and, at higher doses, for obesity. As both conditions become more common worldwide, they raise the risk of serious illness and early death. Semaglutide helps control blood sugar, supports weight loss, and protects the heart, making it an important option for people at risk [8].

Some reports have linked semaglutide to NAION. Simonsen et al. found that people with T2DM who used semaglutide had a higher risk of NAION than those using sodium-glucose cotransporter-2 inhibitors (SGLT2is), though the overall risk is still low [9]. Hsu et al. showed that the risk of NAION increased for semaglutide users at two, three, and four years after starting treatment, especially in patients with both diabetes and high blood pressure. People with diabetes who had used Ozempic (Novo Nordisk) or had a prescription for it also had a higher risk of NAION [10]. Amini et al. found that semaglutide raised NAION rates by 4.28 times in people with type 2 diabetes mellitus and by 7.64 times in those with obesity. NAION usually occurred within 14 months of starting the treatment, whereas cases not related to semaglutide occurred over three years [6]. Hathaway et al. reported that over 36 months, NAION occurred in 8.9% of the semaglutide group and 1.8% of the non-GLP-1 RA group. Their analysis showed a much higher risk of NAION with semaglutide (hazard ratio {HR}, 4.28; P < 0.001). Among overweight or obese patients, 6.7% of semaglutide users developed NAION compared to 0.8% of non-GLP-1 RA users, with a hazard ratio of 7.64 (P < 0.001) [7]. More research is needed to determine whether this link is a direct side effect or related to strict blood sugar control. It is also important to consider both local risk factors, such as a small optic disc and venous congestion, and systemic risk factors, such as diabetes, high blood pressure, high cholesterol, heart disease, stroke, blood clotting problems, irregular heartbeat, anemia, low blood pressure at night, and smoking [7,11].

NAION usually affects those over 50 years old, but it can also occur in younger people with diabetes or other vascular risks. In such patients, medications and metabolic changes may further reduce blood flow to the optic nerve. In this context, the absence of pain, preserved vision and color perception, and the match between optic disc swelling and visual field loss all pointed away from optic neuritis and toward NAION. Furthermore, a thorough evaluation ruled out other diagnoses and confirmed the ischemic nature of the event.

Limitations

This report has several limitations. It focuses on a single patient, lacks a rechallenge, and includes other vascular risk factors such as diabetes. These factors together make it difficult to establish a clear cause-and-effect relationship.

## Conclusions

Clinicians treating patients with diabetes and obesity should remain alert to the possible link between GLP-1 RA therapy and NAION. Monitor patients on GLP-1 RAs, especially semaglutide, for visual symptoms, and arrange prompt ophthalmologic evaluation if symptoms occur. Early detection and intervention are essential to protect visual outcomes and guide timely adjustments to therapy.

Large-scale studies are urgently needed to clarify the relationship between GLP-1 RAs and NAION, identify high-risk individuals, and develop evidence-based monitoring and management guidelines. Until more data are available, implement a multidisciplinary approach involving endocrinologists, ophthalmologists, and primary care providers to ensure patient safety and optimize treatment.
